# P-1036. Invasive fungal Rhinosinusitis in solid organ and hematopoietic stem cell transplant recipients at a tertiary care center in the United States

**DOI:** 10.1093/ofid/ofae631.1226

**Published:** 2025-01-29

**Authors:** Mayyadah Alabdely, Anisha Misra, Deborah Chute, Kyle D Brizendine

**Affiliations:** Cleveland Clinic Foundation, Cleveland, Ohio; Cleveland Clinic Foundation, Cleveland, Ohio; Cleveland Clinic Foundation, Cleveland, Ohio; Cleveland Clinic Foundation, Cleveland, Ohio

## Abstract

**Background:**

Solid organ (SOT) and hematopoietic stem cell transplant (HSCT) recipients are at increased risk of invasive fungal infections. Invasive fungal rhinosinusitis (IFRS) post-transplant has not been well studied due to limited information in large databases. We present the epidemiology of IFRS in SOT and HSCT.

**Table 1:**
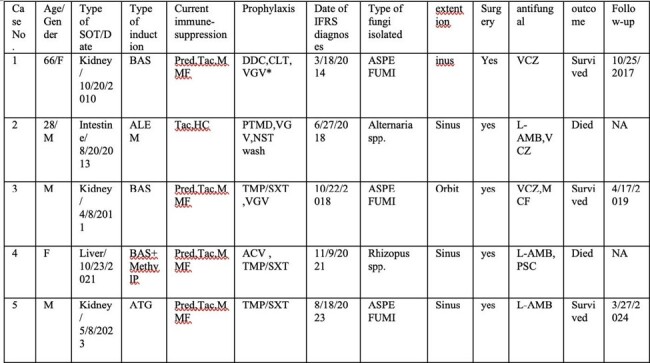
Summary of SOT with invasive fungal rhinosinusitis cases:

**Methods:**

Retrospective study 2010–2022: we identified all cases of IFRS through pathology database. Demographics, organ transplant, immunosuppression, antimicrobial prophylaxis, surgical and medical treatment, and outcomes were examined. IFRS diagnosis was based on histopathological evidence of invasion through the nasal or sinus mucosa or deeper tissue.

**Table 2:**
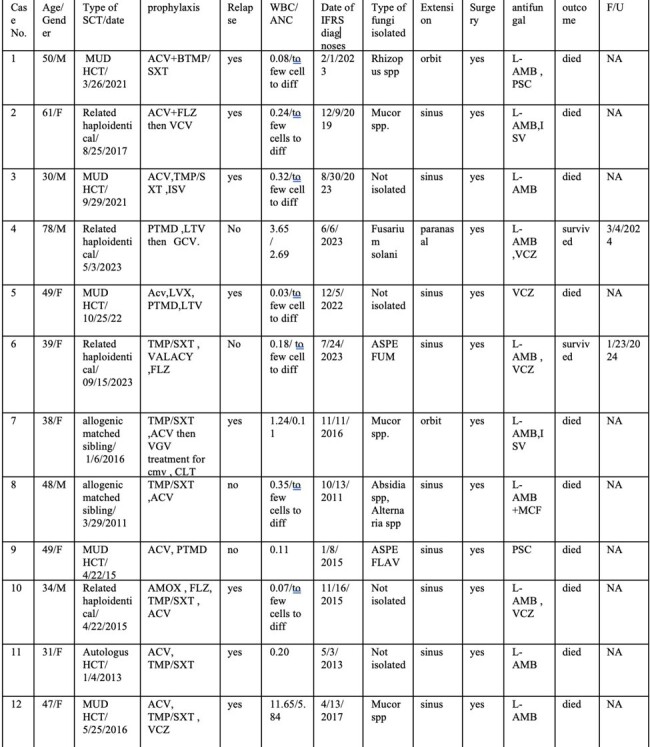
Summary of the hematopoietic stem cell transplant with invasive fungal rhinosinusitis Abbreviations: MMF, Mycophenolate Mofetil; Tac,tacrolimus; Pred,prednisone; BAS, Basiliximab; HC, hydrocortisone; MethylP, Methylprednisolone; ATG, antithymocyte Globulin; DDC, Dapsone; CLT, clotrimazole; VGV, valganciclovir; PTMD, Pentamidine; NST, nystatin; TMP/SXT, trimethoprim–sulfamethoxazole; LVX, levofloxacin; ACV, ayclovir; MCF,Micafungin; ASPE FUMI,Asperigillus fumigatus ; ASPE FLAV,asperigullis flavus; VCZ,Voriconazole; L-AMB,Liposomal Amphoterecin b ; PSC,Posaconazole ; ALEM, alemtuzumab; FLZ, fluconazole; ISV, isavuconazole; AMOX, amoxicillin; VALACY,valacyclovir; LTV,Letermovir ;MUD, matched unrelated donor;HCT, hematopeitic stem cell

**Results:**

Of 101 IFRS cases, 17 were transplant: 5 SOT, 12 HSCT. Mean age for SOT was 55 (range 28–73 years). Organs included 3 kidneys, 1 intestine, 1 liver. Med. time to IFRS after SOT was 1245 d (IQR 102–1772). *Aspergillus fumigatus* was identified in 3 (60%), *Rhizopus* spp. in 1 (20%), and *Alternaria alternata* in 1 (20%). 1 had acute rejection treatment. 2 had CMV viremia. All 5 received surgical and medical interventions. Voriconazole (VCZ) was given to 3 patients along with liposomal amphotericin B (LAmB) and micafungin (Table 1). Mortality was 40%. For HSCT, mean age was 46 (range 30-78 years). 42% HSCT were matched unrelated donor, 25% haploidentical, 25% HLA-matched sibling, and 8% autologous. Med. time to IFRS after HSCT was 254 d (IQR 39–576). Mucormycosis was diagnosed in 4 (33%), *Aspergillus* spp. in 2 (17%), *Fusarium solani* in 1 (8%), and combined *Absidia* spp./ *Alternaria alternata* in 1 (8%); 4 (33%) causative pathogen was not identified. 67% experienced hematologic disease relapse and were undergoing treatment. 25% had CMV. 17% had acute GVHD treatment. 6 (50%) IFRS occurred on antifungal prophylaxis (4 fluconazole, 1 isavuconazole, 1 VCZ). All 12 had surgical and medical management. 10 were treated with LAmB, 1 VCZ, 1 posaconazole (Table 2). Even without intracranial extension, mortality was 83% (Figure 1).

**Figure:**
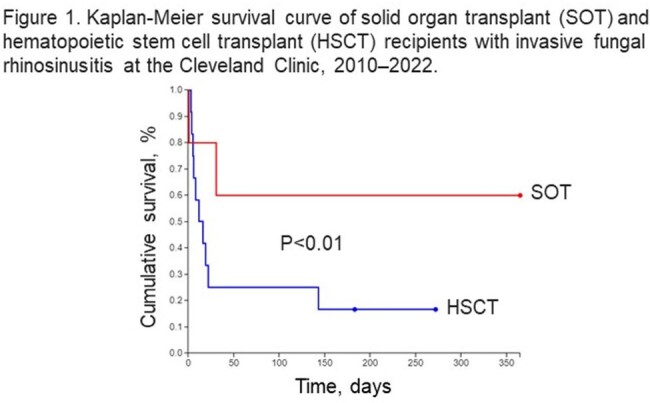

**Conclusion:**

IFRS continues to be associated with high mortality among contemporary patients. Our observations identify important disease differences depending on SOT versus HSCT status. Data analyzing predictors of death and other important outcome measures such as neurologic disability are needed.

**Disclosures:**

**All Authors**: No reported disclosures

